# Mitogen-Inducible Gene-6 Mediates Feedback Inhibition from Mutated BRAF towards the Epidermal Growth Factor Receptor and Thereby Limits Malignant Transformation

**DOI:** 10.1371/journal.pone.0129859

**Published:** 2015-06-12

**Authors:** Malgorzata Milewska, David Romano, Ana Herrero, Maria Luisa Guerriero, Marc Birtwistle, Franz Quehenberger, Stefan Hatzl, Boris N. Kholodenko, Oreste Segatto, Walter Kolch, Armin Zebisch

**Affiliations:** 1 Systems Biology Ireland, University College Dublin, Dublin, Ireland; 2 Icahn School of Medicine at Mount Sinai, New York, New York, United States of America; 3 Institute of Medical Informatics, Statistics and Documentation, Medical University of Graz, Graz, Austria; 4 Division of Hematology, Medical University of Graz, Graz, Austria; 5 Conway Institute of Biomolecular & Biomedical Research, University College Dublin, Dublin, Ireland; 6 School of Medicine and Medical Science, University College Dublin, Dublin, Ireland; 7 Laboratory of Immunology, Regina Elena Cancer Institute, Rome, Italy; Université de Sherbrooke, CANADA

## Abstract

BRAF functions in the RAS-extracellular signal-regulated kinase (ERK) signaling cascade. Activation of this pathway is necessary to mediate the transforming potential of oncogenic BRAF, however, it may also cause a negative feedback that inhibits the epidermal growth factor receptor (EGFR). Mitogen-inducible gene-6 (MIG-6) is a potent inhibitor of the EGFR and has been demonstrated to function as a tumor suppressor. As MIG-6 can be induced via RAS-ERK signaling, we investigated its potential involvement in this negative regulatory loop. Focus formation assays were performed and demonstrated that MIG-6 significantly reduces malignant transformation induced by oncogenic BRAF. Although this genetic interaction was mirrored by a physical interaction between MIG-6 and BRAF, we did not observe a direct regulation of BRAF kinase activity by MIG-6. Interestingly, a selective chemical EGFR inhibitor suppressed transformation to a similar degree as MIG-6, whereas combining these approaches had no synergistic effect. By analyzing a range of BRAF mutated and wildtype cell line models, we could show that BRAF V600E causes a strong upregulation of MIG-6, which was mediated at the transcriptional level via the RAS-ERK pathway and resulted in downregulation of EGFR activation. This feedback loop is operational in tumors, as shown by the analysis of almost 400 patients with papillary thyroid cancer (PTC). Presence of BRAF V600E correlated with increased MIG-6 expression on the one hand, and with inactivation of the EGFR and of PI3K/AKT signaling on the other hand. Importantly, we also observed a more aggressive disease phenotype when BRAF V600E coexisted with low MIG-6 expression. Finally, analysis of methylation data was performed and revealed that higher methylation of MIG-6 correlated to its decreased expression. Taken together, we demonstrate that MIG-6 efficiently reduces cellular transformation driven by oncogenic BRAF by orchestrating a negative feedback circuit directed towards the EGFR.

## Introduction

Somatic mutations within BRAF have been described in a broad range of human tumors, with melanoma, thyroid cancer and colorectal cancer affected most frequently [1–3]. The V600E mutation constitutes the most important alteration conferring high kinase activity and accounting for approximately 90% of BRAF mutations. This mutation has been extensively studied and proved to be a *bona fide* oncogene as evidenced by *in-vitro* and *in-vivo* models [1, 3]. Recently, BRAF inhibitors, such as vemurafenib (PLX4032), have entered the clinical routine [4]. Although vemurafenib achieves high response rates in melanoma, other tumor entities, e.g. colorectal cancer, are rather resistant [5]. Recently, Prahallad and coworkers demonstrated that in colorectal cancer this resistance is caused by the ability of BRAF V600E to induce an inhibitory feedback circuit towards the EGFR [6]. They showed that pharmacologic inhibition of V600E resulted in reactivation of the EGFR, which supported continued proliferation and transformation. While the authors identified CDC25C phosphatase as a potential mediator of this feedback, the potential involvement of additional feedback regulators was not studied in detail. In recent studies of thyroid carcinoma and melanoma, SPRY2 and SOX10 have been identified as additional BRAF-EGFR feedback mediators, which suggests that feedback signaling of BRAF V600E to the EGFR might be more complex than initially thought and includes other, hitherto unknown proteins as well [7, 8].

MIG-6 (also known as ERRFI1, GENE-33 or RALT) is a multiadaptor protein whose expression is induced in response to various stimuli including hormones, multiple growth factors or different cellular stresses [9, 10]. Its transcription is tightly regulated during the cell cycle, which was shown to be mediated predominantly, albeit not exclusively, via the RAS-ERK pathway [9, 10]. MIG-6 is best known for its role as an endogenous inhibitor of EGFR signaling. By associating with an extended surface of the EGFR catalytic domain, MIG-6 locks the receptor in a catalytically inactive configuration and thereby prevents its autophosphorylation [11, 12]. In agreement with this inhibitory function towards the EGFR, MIG-6 has been shown to block the activation of EGFR induced signaling modules, such as the RAS-ERK and PI3K/AKT pathways [11, 13, 14]. In line with this observation, several i*n-vitro* studies indicate that down-regulation of MIG-6 promotes cellular processes regulated by these pathways, including proliferation, migration and invasion. These data suggest a potential tumor suppressor function of MIG-6, which is further strengthened by *in-vivo* studies in murine *Errfi1* knockout models [15–19]. Mice lacking the expression of MIG-6 exhibited prominent hyperactivation of both the EGFR and downstream signaling pathways, as well as spontaneous tumorigenesis in a broad range of tissues [14, 16, 20–24]. In agreement with these observations, a complete or partial loss of MIG-6 expression has been observed in various human malignancies, including carcinomas of the breast, thyroid gland, liver and lungs, as well as in endometrial cancer and glioblastoma [25]. In these studies low levels of MIG-6 correlated with increased phosphorylation of the EGFR, suggesting that in these patients MIG-6 loss promoted tumorigenesis via a defective feedback inhibition of the EGFR.

Recently, MIG-6 expression has been linked to BRAF driven carcinogenesis as patients with V600E positive papillary thyroid cancer (PTC) and low MIG-6 expression demonstrated a more aggressive clinical course as compared to those with high MIG-6 levels [26]. Recent data from melanoma further support this important role in BRAF mediated transformation, as MIG-6 dephosphorylation was associated with EGFR activation and resistance to BRAF inhibitors [27]. In view of these data, we aimed to further investigate a potential role of MIG-6 as negative regulator of BRAF V600E driven transformation and whether this might be orchestrated via an EGFR directed feedback loop.

## Materials and Methods

### Antibodies and Plasmids

The following antibodies were obtained commercially: mouse monoclonal anti-BRAF (Santa Cruz Biotechnology, Dallas, TX), mouse monoclonal anti-RAF1 (BD Transduction Laboratories, Franklin Lakes, NJ), rabbit monoclonal anti-pEGFR (Y1173) (Cell Signaling Technology, Danvers, MA), rabbit polyclonal anti-EGFR (Cell Signaling Technology), mouse monoclonal anti-pERK1/2 (Sigma-Aldrich, St. Louis, MO), rabbit anti-ERK1/2 (Sigma-Aldrich), mouse monoclonal anti-Myc tag (Cell Signaling Technology), IgG from mouse serum (isotype control IgG, Sigma-Aldrich), IgG from rabbit serum (isotype control IgG, Sigma-Aldrich). Mouse monoclonal anti-FLAG M2-Peroxidase (HRP) antibody was purchased from Sigma-Aldrich, rat monoclonal anti-HA-Peroxidase (HRP) antibody was purchased from Roche Diagnostics (Basel, Switzerland). Rabbit polyclonal anti-MIG-6 antibody was either purchased from Santa Cruz Biotechnology or prepared in house [28]. Mammalian expression vectors used in this study were as follows: pcDNA 3.1 (Invitrogen, Carlsbad, CA), pcDNA 3.1 based *HA-MIG-6* vector (encoding hemagglutinin N-terminally tagged to the *MIG-6* coding sequence), pcDNA 3.1 based *GFP-ERK2* and *FLAG-BRAF* plasmid DNA (encoding FLAG tag fused to the C-terminal region of wild type [WT] or the different mutant *BRAF*s), *Myc-MIG-6* and *Myc-EBR*, which are pCS2-MT based vectors (encoding six N-terminal Myc epitopes fused to the *MIG-6* or EGFR Binding Region [EBR] of *MIG-6*). For siRNA experiments, On-Target Plus Smart Pool Human MIG-6 siRNA (GE Dharmacon, Lafayette, CO) and Negative Control siRNA Allstars (Qiagen, Hilden, Germany) were used at a concentration of 60nM.

### Cell Culture and Transfections

African green monkey kidney fibroblast-like cell line (Cos-1), human embryonic kidney cells (HEK293), breast adenocarcinoma (MCF-7), skin epidermoid carcinoma (A431) and mouse embryonic fibroblasts (NIH3T3), human malignant melanoma (A375) and human diploid lung fibroblast cells (WI-38) (obtained from the Kolch laboratory, American Type Culture Collection or European Collection for Cell Cultures) were grown in Dulbecco`s modified minimal essential medium (DMEM) containing 2mM L-glutamine and 10% fetal bovine serum (FBS). All cell lines were screened for authenticity by either variable number of tandem repeat profiling or multipex PCR targeting mitochondrial DNA. To induce quiescence, cell monolayers were rinsed with phosphate-buffered saline (PBS) solution and maintained in the absence of serum for at least 16 hours prior to experimental treatments. For mitogenic stimulations, quiescent cells were treated with 100ng/ml EGF for the specified periods of time. For MEK inhibition experiments, cells were subjected to treatment with 10μM U0126 (Promega, Madison, WI) for 30 minutes. Transient transfections with plasmid DNA were performed using Lipofectamine 2000 (Invitrogen) according to the manufacturer`s instruction. Experiments were conducted 48 hours after transfection.

### Cell Transformation Assay

Early passage NIH3T3 clone seven cells were split at very low confluency into 10cm dishes. Twenty-four hours later cells were transiently transfected with 400ng plasmid DNA of the appropriate constructs using Lipofectamine 2000 (Invitrogen). Six hours post-transfection the medium was changed into DMEM containing donor bovine serum (Invitrogen). Subsequently, cells were grown until foci became macroscopically visible, fixed using 100% methanol and stained for one hour with 5% Giemsa solution as previously described [29]. Transformed foci were counted, and the average number of foci per ng of transfected DNA was expressed as fold difference to an untransfected control. To study the effect of EGFR inhibition on focus formation, cells were treated with 3μM BIBX1382 (Merck, Darmstadt, Germany) after transfection, which proved to be a saturating concentration in dilution experiments.

### Western blotting

Whole cell lysates were prepared by washing cells with PBS followed by lysis in ice-cold lysis buffer (20mM Hepes-KOH [pH 7.5], 150mM NaCl, 1% NP-40, protease and phosphatase inhibitor cocktail (Thermo Scientific, Waltham, MA). Protein extracts were clarified by centrifugation, resolved by SDS-PAGE using NuPage Bis-Tris gels (Invitrogen), and transferred to PVDF membranes. Proteins were detected by chemiluminescence (Thermo Scientific) using standard protocols. Protein band intensity was quantified using digital image densitometry analysis using the ImageJ software (National Institutes of Health, Bethesda, MD).

### Immunoprecipitation

For immunoprecipitation (IP) of endogenous proteins and the Myc-tag, respectively, total protein extracts were prepared as described above and pre-cleared with bovine serum albumin (BSA) pre-blocked Protein A-Sepharose 4B or Protein G-Sepharose Fast Flow (Sigma-Aldrich) for one hour at 4°C. Subsequently, lysates were mixed with the appropriate antibody or IgG control for two hours at 4°C by rocking followed by incubation with Protein A or Protein G conjugated beads for additional 1 hour and 30 minutes at 4°C. For IP of endogenous proteins, we used 2μg of anti-BRAF or anti-MIG-6 antibody and 2μg of appropriate control IgG, while for anti-Myc IPs we used anti-Myc Ab (1:500). For FLAG IPs, cell lysates were combined with 20μl of FLAG beads (ANTI-FLAG-M2 Affinity Gel [Sigma-Aldrich]) or 20 μl of GFP beads (ChromoTek, Martinsried, Germany) respectively, and rotated for three hours at 4°C. The protein concentration was 200μg in all conditions. All IPs were washed with lysis buffer three times, suspended in 2X NuPage LDS Sample Buffer (Invitrogen) supplemented with β-mercaptoethanol (2.5% final concentration), and incubated for 10 minutes at 70°C. Samples were resolved on NuPage Bis-Tris gels (Invitrogen) and blotted.

### In-vitro Kinase Assay

To perform *in-vitro* kinase assays, Cos-1 cells were transfected with FLAG-BRAF WT and FLAG-BRAF V600E, respectively, with and without co-transfection using an HA-MIG-6 expression vector. After 48 hours of incubation, cells were washed twice in ice-cold PBS and lysed by scraping into ice cold RIPA buffer (20mM Tris-HCl [pH. 7.5], 150mM NaCl, 1% Triton X-100, 0.5% sodium deoxycholate, protease and phosphatase inhibitor cocktail [Thermo Scientific]). Next, pre-cleared protein extracts were subjected to immunoprecipitation using ANTI-FLAG-M2 Affinity Gel (Sigma-Aldrich) for three hours at 4°C. Precipitates were washed four times with washing buffer (RIPA buffer without sodium deoxycholate), followed by three gentle washings with kinase assay buffer (50mM Tris-HCl [pH. 7,5], 75mM NaCl, 5mM EGTA, 5mM Mg_2_Cl, 1mM DTT and 1mM sodium orthovanadate). Each immunoprecipitate was then incubated with 100ng recombinant WT GST-MEK1 (Millipore, Darmstadt, Germany), 1μg kinase dead ERK2 (K54R) (ProQinase, Freiburg, Germany) and 100μM ATP at 30°C for 20 minutes. Reactions were resolved on 4–12% NuPage Tris-Bis gels (Invitrogen), and ERK phosphorylation was assessed by immunoblotting with anti-pERK1/2 (Sigma-Aldrich).

### MIG-6 qPCR expression analysis

Total RNA of A431 cells was extracted using TRIzol-Reagent (Ambion, Life-Technologies, Carlsbad, CA) according to the manufacturer´s instructions. All samples were diluted to a final concentration of 100ng/μL total RNA. Reverse transcription of RNA was done with 0,5μg of RNA using the TaqMan-Reverse Transcription Reagents (Applied Biosystems, Foster City, CA; manufactured by Roche, Branchburg, NJ) according to the protocol of the manufacturer and cDNA was stored at -20°C until further use. Expression analysis was done on an ABI Prism 7000 Sequence Detection System using the SYBR Green method (Invitrogen Life-Technologies, Carlsbad, CA) and *MIG-6* cDNA expression was evaluated using the comparative ddCT method as previously described [29–31]. A431 cells transfected with empty vector served as a calibrator. *RPL37A*, *GAPDH* and *GUSB* were used as endogenous control genes, which was based on the fact that they were previously shown to be amongst the most stable control genes within this cell line [32]. Primers for *MIG-6* and endogenous control genes were generated via PrimerBank (http://pga.mgh.harvard.edu/primerbank/) [33] and are listed in S1 Table. All primers were checked for specifity using the BLAST software (http://blast.ncbi.nlm.nih.gov/Blast.cgi) and PCR efficiency was evaluated before expression analysis was started. Amplifications were done at ABI Prism 7000 standard conditions as previously described [29–31]. Primer concentrations used were 10pmol for all primers.

### Evaluation of Primary Patient Samples and Statistical Analyses

Clinical data of PTC were downloaded from The Cancer and Genome Atlas (TCGA, www.cancergenome.nih.gov) on 8 January 2014, on 8 September 2014 (EGFR data) and on 31 March 2015 (MEK, ERK, AKT, mTOR and MIG-6 methylation data). Risk stratification was performed according to the American Joint Commission on Cancer-International Union Against Cancer (AJCC/IUAC) criteria [34]. Briefly, patients younger than 45 years, who had pTNM stage I as well as patients aged 45 or older, who had stage I or II were defined as low-risk group. All other patients (stage II in patients <45 years and stage III or IV in patients ≥45 years) were defined as high-risk group. BRAF mutational status (as obtained by whole exome sequencing), *MIG-6* gene expression (as obtained by RNA Sequencing V2 RSEM) and methylation (as obtained by Infimum Human Methylation HM450) as well as MEK1/2 S217/S221, ERK T202/Y204, EGFR Y1173, ERBB2 Y1248, AKT (T308 and S473) and mTOR S2448 phosphorylation and MIG-6 protein expression (all obtained by reverse phase protein array) within this cohort were analyzed using the cBioPortal for Cancer Genomics (www.cbioportal.org/public-portal/index.do) [35, 36]. A comparison of *MIG-6* mRNA expression between samples with and without BRAF V600E as well as between high-risk and low-risk patients was performed using the Mann-Whitney-Wilcoxon test. The same test was applied for comparison of phospho-protein level z-scores between samples with and without BRAF V600E. To compare the frequency of BRAF mutations between risk stages, a Fisher´s exact test was employed. For correlation of MIG-6 expression and methylation, Spearman-Rho and Pearson correlation coefficients were calculated. For analysis of *in-vitro* experiments, Student’s t-test was calculated from at least three independent experiments. R 3.0.2 (www.r-project.org) and Graph Pad Prism (http://www.graphpad.com/scientific-software/prism/) were used for analysis. All tests were performed two-sided and a P-value of < 0.0500 was considered statistically significant.

## Results

### MIG-6 Inhibits Cellular Transformation Induced by Mutant BRAF via Inhibition of the EGFR

**Fig 1:**
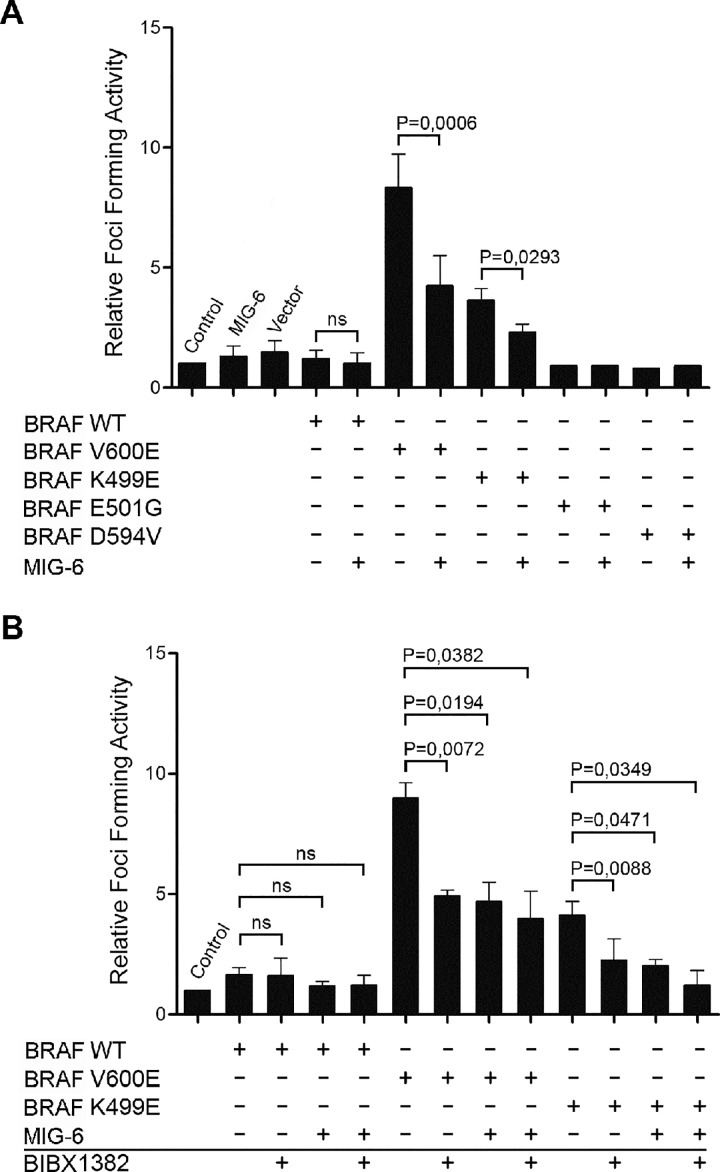
MIG-6 Inhibits Cellular Transformation Driven by Mutations in BRAF. **A.** For focus formation assays, NIH3T3 cells were transiently transfected with the indicated constructs (400ng *BRAF*; 400ng *MIG-6/empty vector*) and cultivated until foci became macroscopically visible. Untransfected cells were included as an additional control. Foci were stained with Giemsa and counted. Data show the fold change of the average number of foci per ng of transfected DNA over untransfected control cells. **B.** Focus formation was evaluated as described above, except that after transfection cells were cultured in the presence of a specific EGFR inhibitor (3μM BIBX1382) as indicated. Graphs denote the mean ± standard deviation (SD) from three independent experiments. P-values were calculated using paired Student’s t-test. (ns, non-significant).

A variety of BRAF mutations, including the most frequent V600E substitution, have been demonstrated to induce malignant transformation [2]. To test for a potential involvement of MIG-6 in this process, we studied the effects of MIG-6 overexpression in a series of BRAF driven focus formation assays, which allow the quantification of the transforming strengths of BRAF [37, 38]. NIH3T3 were transiently transfected with plasmids encoding FLAG-tagged versions of either WT or mutant (V600E, K499E, E501G, D594V) BRAF. As expected, BRAF V600E potently induced cellular transformation (Fig 1A). K499E, which has a weakly elevated kinase activity, still caused a moderate increase in focus formation, whereas BRAF WT and kinase impaired mutants (E501G and D594V) failed to induce foci (Fig 1A). Surprisingly, co-transfection of an HA-tagged *MIG-6* expression construct significantly decreased the number of foci formed by both the BRAF V600E and K499E mutants, indicating that MIG-6 effectively inhibits BRAF mediated cellular transformation (Fig 1A). After having proven that the NIH3T3 cells used in this study respond to EGFR inhibition (S1 File), we treated transfected cells with the selective EGFR inhibitor BIBX1382 [39]. Interestingly, EGFR inhibition markedly reduced the focus forming ability of both BRAF V600E and K499E (Fig 1B), suggesting that oncogenic BRAF requires at least some EGFR kinase activity for full transformation. The extent of inhibition induced by BIBX1382 was comparable to the effect of MIG-6 introduction. Importantly, simultaneous MIG-6 transfection and BIBX1382 treatment did not cause further inhibition of transformation, an observation suggesting that MIG-6 antagonizes mutant BRAF transformation via EGFR inhibition. Taken together, these results indicate that MIG-6 can inhibit transformation elicited by mutant BRAF, and that this inhibitory effect is likely due to the inhibition of the EGFR. Therefore, we examined potential mechanisms for how MIG-6 could exert this role.

### MIG-6 and BRAF are Novel Interaction Partners

**Fig 2:**
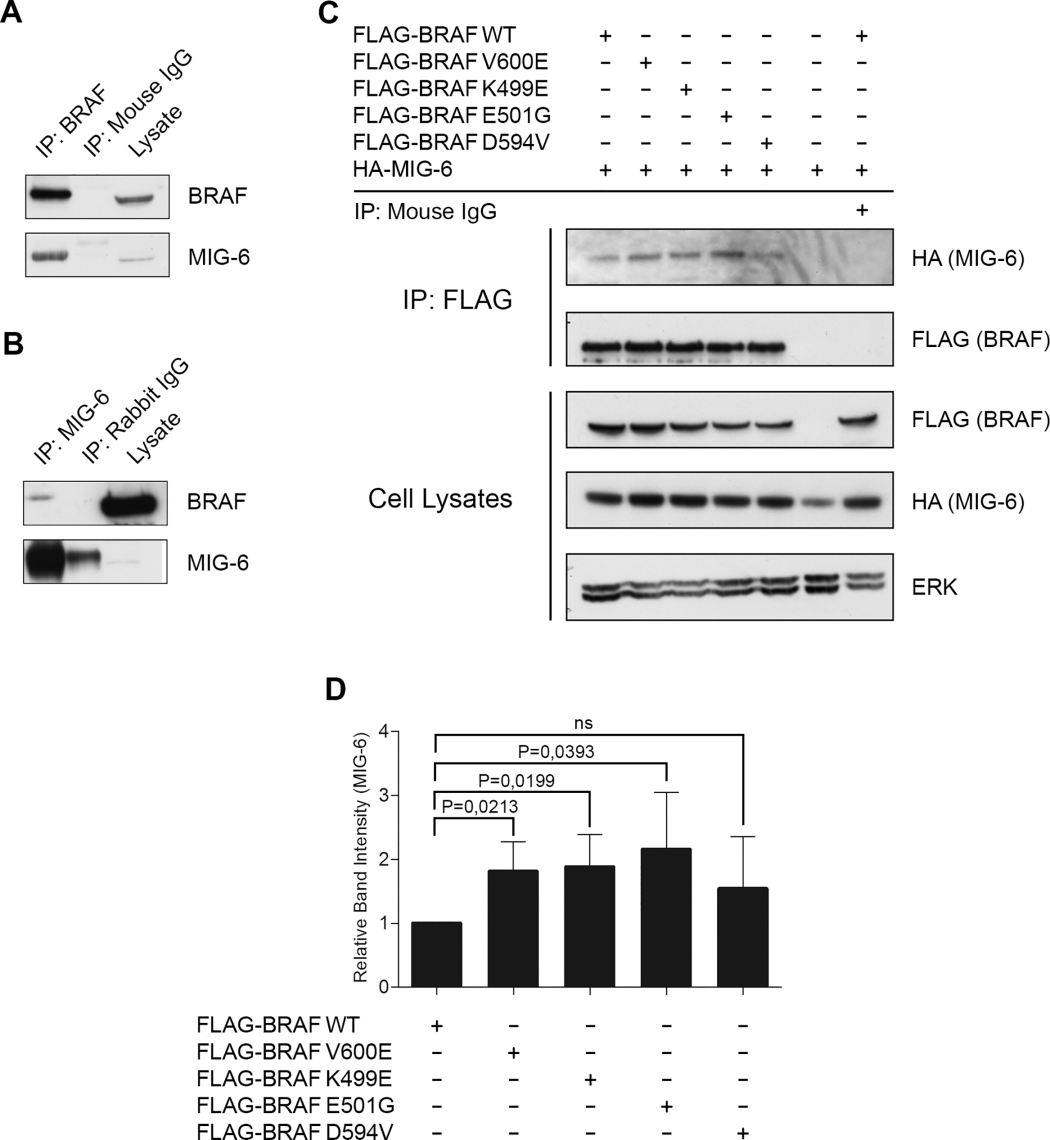
MIG-6 Interacts with BRAF. **A.** Cos-1 total cell extracts were immunoprecipitated using an anti-BRAF antibody and immunoblotted with the indicated antibodies. **B.** Cellular extracts from Cos-1 were immunoprecipitated with anti-MIG-6 antibody followed by Western blot analysis detecting BRAF and MIG-6, respectively. **C.** Cos-1 cells were transiently transfected with plasmid DNA encoding FLAG fused to WT or mutant *BRAF* and co-transfected with *HA-MIG-6*. Whole cell extracts were subjected to anti-FLAG specific IP and blotted with the indicated antibodies. **D.** Densitometrical analysis of MIG-6 band intensity in anti-FLAG specific IPs. MIG-6 co-immunoprecipitation is reported as changes normalized to MIG-6 co-immunoprecipitating with FLAG-BRAF WT. Data represent the mean of three independent experiments ± SD. P values were calculated using paired Student`s t-test.

In a proteomics screen in PC12 cells (rat adrenal pheochromocytoma) we previously identified MIG-6 as a potential binding partner of BRAF WT. To confirm these preliminary data, we performed co-immunoprecipitation experiments between endogenous BRAF and MIG-6 proteins in Cos-1 cells. MIG-6 co-immunoprecipitated with BRAF (Fig 2A), and BRAF co-immunoprecipitated with MIG-6 (Fig 2B), confirming that endogenous MIG-6 and BRAF are indeed interacting proteins. Unfortunately, antibodies used for IgG control and MIG-6 were both derived from rabbit, which might explain the additional band in Fig 2B. Of note, our data showed that a rather large fraction of MIG-6 interacts with BRAF, whereas only a minor fraction of BRAF interacts with MIG-6. This phenomenon might be explainable by the fact that BRAF expression in Cos-1 cells is vastly more abundant than MIG-6, however, the possibility that anti-MIG-6 antibodies could react against an epitope close to the BRAF binding site, causing either inefficient IP of BRAF-bound MIG-6 molecules or displacement of BRAF cannot be excluded. We next evaluated whether this interaction is influenced by the presence of mutations in BRAF. For this purpose we co-transfected Cos-1 cells with HA-tagged MIG-6, FLAG-labeled WT or BRAF mutants that span a range of kinase activities, i.e. V600E and K499E having elevated activities; E501G having activity similar to WT BRAF, and D594V having activity lower than WT BRAF [40–42]. BRAF was immunoprecipitated using an anti-FLAG antibody and the presence of MIG-6 protein was assessed by Western blots using an antibody specific for the HA-epitope (Fig 2C). MIG-6 can bind to both WT and mutant BRAF, as evidenced by the fact that MIG-6 was detected in all conditions tested. Densitometric quantification of the blots revealed that MIG-6 preferentially interacted with BRAF mutants that have elevated or normal kinase activity (Fig 2D). To exclude the possibility that the interaction between BRAF and MIG-6 were biased due to a potential Cos-1 specific effect, we additionally confirmed the association between MIG-6 and BRAF in additional cell lines (MIG-6/BRAF WT in WI-38 human lung fibroblasts and MIG-6/BRAF V600E in A375 cells; S2 File). Of note, we did not observe interaction between MIG-6 and ERK as previously suggested by Kim and coworkers in endometrial cancer [43]. It is unclear whether this is due to differences in experimental conditions or cellular context. To further test for functional consequences of this interaction, we tested whether MIG-6 influences the kinase activity of BRAF in a first step. For this purpose, we analyzed *in-vitro* kinase activities of BRAF/MIG-6 immunoprecipitates after co-transfection of HA-tagged MIG-6 and FLAG-tagged BRAF but failed to observe any effects (S3 File). As BRAF activity can also be regulated by heterodimerization with RAF1, we next investigated whether MIG-6 plays a role in the BRAF/RAF1 heterodimerization process. Of note, however, we failed to observe any significant influence on BRAF/RAF1 dimerization (S4 File).

### MIG-6 Binds Independently to BRAF and the EGFR

**Fig 3:**
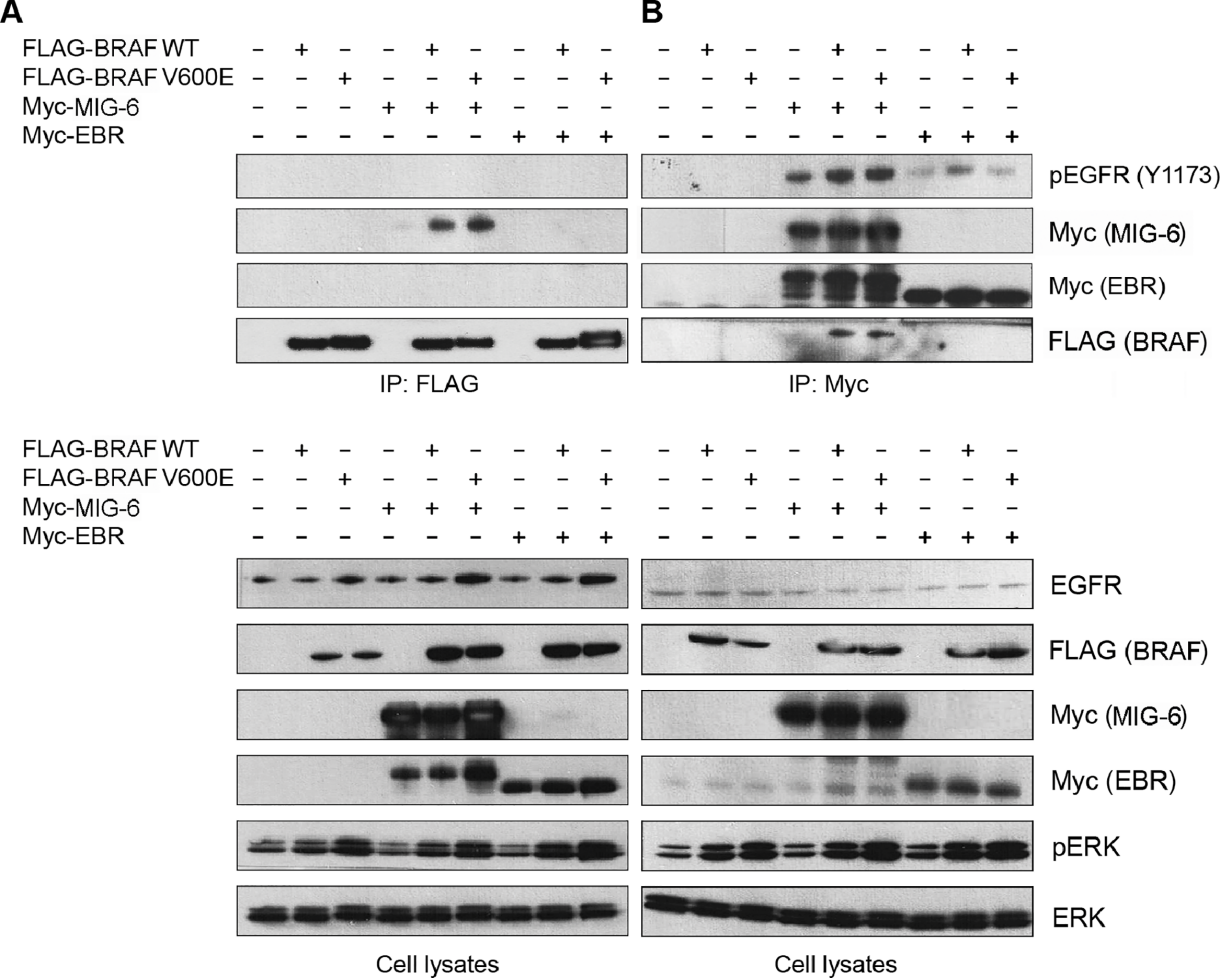
MIG-6 Binds Independently to BRAF and the EGFR. Cos-1 cells were transiently co-transfected with FLAG-tagged *BRAF* WT and V600E, together with either Myc-tagged *MIG-6* or *EBR* (ErbB binding domain of MIG-6). **A.** Anti-FLAG immunoprecipitates (upper panel) as well as total cell extracts (lower panel) of serum starved cells were immunoblotted with the indicated antibodies and demonstrate the interaction between BRAF and full length MIG-6 (lanes 4–6) but not EBR (lanes 7–9). **B.** Reciprocal anti-Myc immunoprecipitates (upper panel) and total cell extracts (lower panel) of serum starved cells were subjected to Western blot analysis with the indicated antibodies, again showing interaction between BRAF and full length MIG-6 only (lanes 4–6).

MIG-6 is predominantly known for its role as a negative regulator of the EGFR family. Hence, we tested whether binding of MIG-6 to BRAF affects its interaction with this receptor. For this purpose, we transiently transfected Cos-1 cells with FLAG-tagged BRAF WT and V600E expression vectors, either alone or in combination with Myc-tagged MIG-6 and EBR constructs, respectively. EBR (also known as ErbB binding domain of MIG-6) is a fragment of MIG-6, spanning the region from amino acid 284–399 and has been shown to be the minimal region of MIG-6, that can be expressed in mammalian cells at a satisfactory level and that still binds to the EGFR [11]. In serum starved cells, BRAF proteins co-immunoprecipitated with full length MIG-6 but not with EBR (Fig 3A). This observation could be corroborated in reciprocal immunoprecipitations using anti-Myc (Fig 3B), indicating that the EGFR binding domain of MIG-6 is not involved in binding BRAF. Of note, these findings were reproducible after FBS incubation, thereby excluding an influence of growth factor stimulation. To analyze whether the interaction between MIG-6 and BRAF influences the interaction between MIG-6 and the EGFR, we stained the anti-FLAG and anti-Myc specific IPs with a pEGFR (Y1173) antibody that recognizes the major autophosphorylation site of the EGFR. Interestingly, pEGFR specific bands were easily detected in the anti-Myc, i.e. MIG-6 and EBR, immunoprecipitates, but not in the BRAF immunoprecipitates. These results suggest that there are no ternary complexes between the EGFR, MIG-6 and BRAF, but that MIG-6 interacts with BRAF and the EGFR in separate complexes. As outlined above, however, MIG-6 did not impact on BRAF V600E kinase activity (S3 File) or BRAF/RAF1 heterodimerization (S4 File).

### Oncogenic BRAF V600E Induces MIG-6 Expression, which Results in a Negative Feedback towards the EGFR

**Fig 4:**
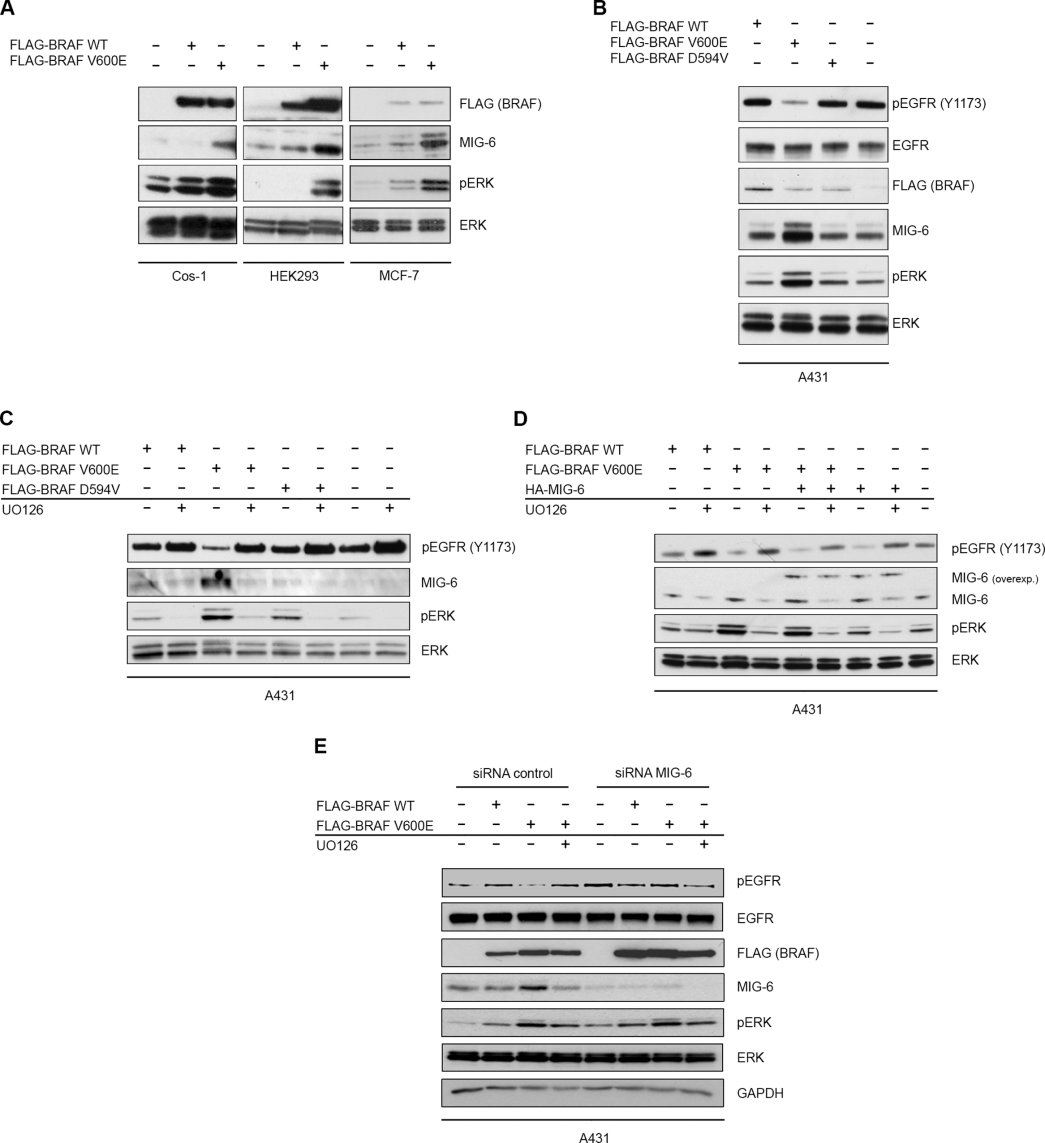
Oncogenic BRAF V600E Induces Expression of Endogenous MIG-6, which in turn Negatively Regulates the EGFR. **A.** Cos-1, HEK293 and MCF-7 cell lines were transiently transfected with *FLAG-BRAF* WT or V600E expression plasmids. Total cell extracts were analyzed by Western blotting with the indicated antibodies. **B.** A431 cells were transiently transfected with expression vectors for BRAF WT, V600E or the impaired kinase activity mutant D594V. Total cell extracts were analyzed by immunoblotting with the depicted antibodies. **C**,**D.** The same experiment as in B except that treatment with 10μM U0126 MEK inhibitor for 30 minutes (panel C—lanes 2, 4, 6 and 8; panel D—lanes 2, 4, 6 and 8) and/or MIG-6 overexpression (panel D—lanes 5–8) was included. **E.** A431 cells were transiently transfected with *FLAG-BRAF* WT or V600E expression plasmids as indicated. Treatment with the MEK inhibitor U0126 (lanes 4 and 8) as well as concomitant transfection with either control (lanes 1–4) or MIG-6 siRNA (lanes 5–8) were included as additional conditions.

We demonstrate that MIG-6 inhibits BRAF driven malignant transformation and describe MIG-6 as a novel interaction partner of BRAF. Importantly, however, MIG-6 failed to regulate BRAF kinase activity and BRAF/RAF1 heterodimerization in our experiments. Therefore, the inhibitory effect of MIG-6 on BRAF transformation must rely on a different mechanism. As shown in Fig 1, we observed inhibition of BRAF-driven transformation by the EGFR inhibitor BIBX1382 as well, which suggested that EGFR activity is required for cell transformation mediated by BRAF V600E. A negative feedback loop from ERK to the EGFR has already been proposed previously in colon cancer cell lines with mutant BRAF, where ERK phosphorylation induced the activation of a CDC25C phosphatase, which in turn can dephosphorylate and inhibit the EGFR [6]. Hence, we tested whether MIG-6 could mediate additional feedback inhibition of the EGFR in cells expressing BRAF V600E. In order to examine how the presence of oncogenic BRAF V600E affects the expression levels of endogenous MIG-6, we transfected different cell lines with expression vectors encoding either FLAG-tagged BRAF WT or V600E. Transient overexpression of BRAF V600E resulted in a strong increase of endogenous MIG-6 in all cell lines studied (Fig 4A). To further study the impact of BRAF V600E on the activation status of the EGFR, we employed the A431 cell line, which expresses the endogenous EGFR at high levels allowing easier detection of pEGFR by Western blotting [44]. Transfection of the high kinase activity mutant FLAG-BRAF V600E, but not of WT BRAF or a kinase impaired mutant (D594V) caused activation of ERK and increased endogenous MIG-6 expression (Fig 4B). Most importantly however, cells carrying the BRAF V600E mutation demonstrated a strongly decreased level of phospho-EGFR (Fig 4B) as compared to the WT and the kinase impaired BRAF mutant. To prove that these effects are truly mediated via ERK signaling, we transiently transfected A431 cells with FLAG-BRAF WT, V600E or D594V expression vectors, and subsequently treated the cells with the pharmacological MEK inhibitor, U0126. U0126 resulted in a profound decrease of endogenous MIG-6 expression, as well as in a global increase of phosphorylated EGFR levels (Fig 4C). This effect was most pronounced in the cells transfected with the high activity BRAF V600E mutant (Fig 4C, lane 3 and 4). To further delineate whether BRAF V600E induces MIG-6 expression at the transcriptional or post-transcriptional level, we repeated these experiments and performed qPCR analyses of *MIG-6* expression as previously reported [29–31]. Importantly, *MIG-6* mRNA was significantly increased after BRAF V600E transfection pinpointing to a transcriptional regulation (S5 File). ERK activation induces several negative feedback regulators of EGFR signaling including SPRY2, SPRED, and others [45]. Therefore, it was important to test whether the BRAF V600E induced EGFR inhibition was due to MIG-6 induction. For this purpose, we repeated the U0126 experiment described above, but included the transfection of HA-MIG-6 as an additional condition. Expression of HA-MIG-6 increased the total amount of MIG-6 protein which resulted in an additional decrease of active pEGFR (Fig 4D), indicating that the V600E induced feedback inhibition of the EGFR via MIG-6 relies on the total amount of MIG-6 expression. Importantly, exogenous MIG-6 expression was not affected by MEK inhibition and still functioned to inhibit the EGFR (Fig 4D). It has to be noted, however, that phosphorylated EGFR levels still remained some sensitivity to U0126 after transfection of MIG-6. This might be explainable by the fact that the effects of MIG-6 on pEGFR rely on total MIG-6 expression (which is the sum of exogenous and the U0126-regulated endogenous MIG-6). Additionally, it might pinpoint to additional, MIG-6 independent feedbacks which directly signal from ERK the EGFR, as previously shown by Red-Brewer and coworkers [46]. In this case, MIG-6 transfection and U0126 would synergistically act to inhibit the EGFR. To further prove the seminal and specific role of MIG-6 within this feedback, we repeated the transfection of BRAF WT and V600E in presence of MIG-6 silencing by means of siRNA. Importantly, BRAF V600E induced the expression of MIG-6 and the downregulation of phosphorylated EGFR when cotransfected with control siRNA, but failed to do so when specific MIG-6 siRNA was used (Fig 4E). Finally, we were interested whether this mechanism applies for BRAF mutated cancer cell lines as well and therefore analyzed *MIG-6* expression in previously published microarray data, obtained within the BRAF V600E mutated melanoma cell line A375 [47]. In agreement with our transfection experiments, specific BRAF V600E inhibition by vemurafenib caused a significant decrease in *MIG-6* expression (S6 File). Taken together, these results indicate that MIG-6 is the BRAF-ERK induced feedback inhibitor responsible for suppression of EGFR phosphorylation.

### Decreased Expression of MIG-6 is Associated with a More Aggressive Phenotype of BRAF Mutated Papillary Thyroid Cancer

**Fig 5:**
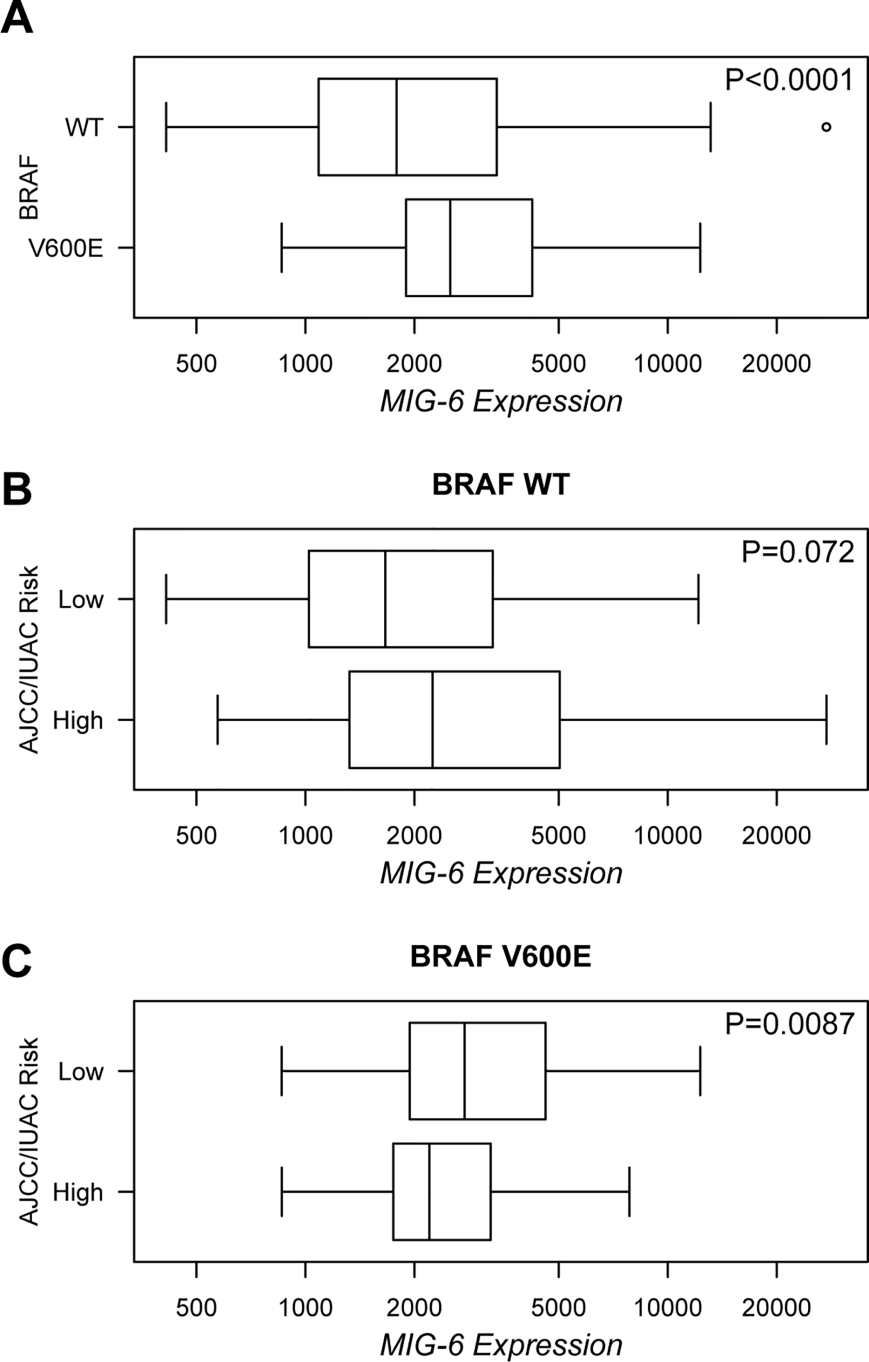
*MIG-6* Expression is Increased in V600E Mutated PTC and Enables Risk Stratification of these Patients. **A.** Box plot showing a significant increase of *MIG-6* mRNA expression (displayed as RNA Sequencing V2 RSEM expression values at the x-axis) in PTC specimens carrying a BRAF V600E mutation as compared to those with BRAF WT. **B**,**C.** Box plots demonstrating the comparison of *MIG-6* expression (values displayed as in panel A) between AJCC/IUAC risk groups in BRAF WT (B) and V600E mutated (C) patients. Data of PTC patient samples (n = 392) were downloaded from the TCGA portal and analyzed using the cBioPortal for Cancer Genomics. P-values were calculated using Mann-Whitney-Wilcoxon test.

In colon cancer the disruption of the negative feedback loop from ERK to the EGFR confers resistance to RAF inhibitor drugs and promotes transformation despite RAF inhibition [6]. As recently suggested by Girotti and coworkers, our results indicate that MIG-6 can exert a negative feedback from ERK to the EGFR (Fig 4), and that MIG-6 expression is equipotent to EGFR inhibition in *in-vitro* transformation assays (Fig 1) [27]. Thus, we were interested to investigate whether these findings are relevant to BRAF driven human carcinogenesis. We focused on PTC, as the BRAF V600E mutation is a frequent genetic alteration in PTC, with frequencies of up to 73%, and as previous studies had indicated a potential tumor suppressor function of MIG-6 in this disease [17, 48, 49]. We downloaded a set of 392 PTC cases from The Cancer Genome Atlas (TCGA) and analyzed the BRAF V600E mutation status, *MIG-6* mRNA expression levels, and the AJCC/IUAC risk category, respectively. In order to examine a possible correlation between *MIG-6* expression levels and mutated BRAF V600E, we analyzed samples where information on both *BRAF* mutation status and *MIG-6* mRNA expression levels was available (n = 381). 236 out of 381 (61.9%) specimens exhibited mutations in *BRAF*, with the V600E substitution accounting for 95.8% of the cases. As we were specifically interested in the effects of the V600E mutation we excluded other mutations, and compared *MIG-6* expression levels between WT (n = 145) and BRAF V600E mutated (n = 226) cases. In agreement with the results obtained in our cell line models, *MIG-6* expression showed a highly significant increase in PTC samples harboring the BRAF V600E mutation as compared to the cases with BRAF WT (P < 0.001; Fig 5A). We then assessed whether increased *MIG-6* expression in BRAF V600E mutated PTC patients correlates to activation of the RAS-ERK pathway and to downregulation of EGFR phosphorylation. Therefore we analyzed phospho-levels of MEK1/2 (S217/S221) and ERK1/2 (T202/Y204), as well as of EGFR (Y1173) and ERBB2 (Y1248), which have been shown to be direct targets of MIG-6 [13, 24, 50]. Although data entries were available for 197 of 381 (51.7%) patients only, phosphorylation levels were significantly increased for MEK1/2 (P = 0.0011) and decreased for both EGFR (P < 0.001) and ERBB2 (P < 0.001) in PTC patients carrying a BRAF V600E mutation, which further supports our *in-vitro* data (S7 File). Of note, phospho-levels of ERK1/2 were not significantly increased in this data set, which is in contrast to our findings in MEK. While this discrepancy might be caused by the smaller sample size with phosphorylation data available or the quality of the ERK dataset, future studies specifically addressing this issue will definitely be needed. Unfortunately, phosphorylation data for ERBB3, which has been connected to BRAF feedback signaling in PTC recently [51] were not available within this data set. To further prove the downregulation of EGFR phosphorylation, we additionally checked for inactivation of the PI3K/AKT pathway, another EGFR mediated signaling cascade, by analyzing the phosphorylation status of AKT (T308 and S473) and mTOR (S2448). Indeed, the increase of *MIG-6* expression in BRAF V600E mutated tumors significantly correlated to a decreased activation status of both AKT (P = 0.0005 for T308 and P = 0.0020 for S473) and mTOR (P = 0.0021; S8 File).

Next, we examined whether *MIG-6* expression is associated with the clinicopathologic phenotype of PTC, and therefore performed risk stratification according to the AJCC/IUAC system. This model includes the pathologic TNM stage as well as the age at diagnosis, and is the most commonly used model for risk assessment in PTC [34]. Analyzing cases with information on BRAF mutational status, *MIG-6* expression and AJCC/IUAC risk stratification, high risk cases were identified in 121 of 379 (31.9%) patients. As described previously, BRAF V600E was significantly associated with a higher risk stage (P < 0.001); 22.2% of WT and 38.7% of V600E mutated patients were classified within this category [52]. We then analyzed *MIG-6* expression within this context. Interestingly, low *MIG-6* expression correlated with a higher AJCC/IUAC risk stage in patients exhibiting a V600E substitution (P = 0.0087; Fig 5B and 5C), whereas no correlation could be observed in the total cohort and in BRAF WT patients. A trend to the same phenomenon was observed when lymph node metastases were analyzed (N0 vs N1), although statistical significance was just not reached in this case (P = 0.0600 for V600E and P = 0.1870 for WT). It has to be noted though, that information of the lymph node status was available for a subset of patients only, which might have biased this analysis. Unfortunately, the phosphorylation status of EGFR and ERBB2 could not be analyzed within this context, as the small sample size of groups with all data entries available precluded proper statistical testing. Although these data have to be considered as preliminary, they point towards a specific inhibitory role of MIG-6 in BRAF V600E mutated PTC, as suggested in our *in-vitro* data. Consequently, one might speculate that secondary, (epi-) genetic downregulation of MIG-6 facilitates an increased BRAF oncogenic potential and the development of more aggressive tumors. To further study this possibility, we studied whether high risk tumors lack BRAF V600E induced *MIG-6* overexpression. Indeed, while BRAF V600E correlated to increased *MIG-6* expression in AJCC/IUAC low risk cases (P < 0.0001), it failed to do so in high risk patients (P = 0.2830). The same phenomenon was observed when comparing cases with and without lymph node metastases (P < 0.0001 for N0 cases; P = 0.1130 for N1 cases). As the TCGA platform now also provides methylation data, we tried to evaluate whether decreased expression of *MIG-6* might correlate to its increased methylation within this cancer entity as previously suggested [17]. In agreement with this study, we observed a statistically significant inverse correlation between *MIG-6* expression and methylation within PTC (P < 0.0001; S9 File). As outlined above, these data have to be considered as preliminary, however, they might provide the basis for the design of experiments using the future generation of *in-vivo* PTC models, combining a knockout of Mig-6 with mutations in Braf, as well as for prospective clinical trials.

## Discussion

MIG-6 is a direct and physiologic inhibitor of the EGFR family. Its expression is induced by various mitogenic signals, usually through stimulation of *MIG-6* gene transcription, thereby generating a negative feedback loop that may serve to temporally limit physiological EGF stimulation. A tumor suppressor function for MIG-6 has been proposed based on experimental observations obtained in a broad range of *in-vitro* and *in-vivo* model systems. Most importantly, however, a pathologic decrease in MIG-6 expression has been detected in several human carcinomas, which could be linked to aberrant EGFR signaling and a more aggressive disease course [25]. In this study, we wanted to investigate a potential role of MIG-6 in restraining BRAF V600E mediated transformation. Initially, we identified MIG-6 as a novel interaction partner of BRAF and demonstrated increased affinity to a range of BRAF mutations. As this observation is based on IP experiments, we cannot exclude the possibility that BRAF and MIG-6 are rather part of the same complex than true binding partners. In any case, we did not observe any effects on BRAF kinase activity and heterodimerization with RAF1. These data rule out a relevant role of the MIG-6/BRAF physical interaction in regulating BRAF oncogenic activity. We therefore focused our attention on the role of MIG-6 in EGFR regulation, given that pharmacological inhibition of EGFR was equipotent and non-additive to MIG-6 in curbing cell transformation mediated by BRAF V600E. This suggested that a) oncogenic BRAF requires at least some EGFR kinase activity to fully exert its oncogenic effects, and b) MIG-6 might restrain BRAF V600E transformation by acting in a negative feedback circuit that targets the EGFR (and possibly other ErbB receptors). *In-vitro* data, obtained from different cell line models with BRAF WT and V600E, as well as data acquired from PTC patients further support the existence of such an inhibitory loop and the pivotal role of MIG-6 within this process. One might speculate, that such a negative feedback influences the activity of other, ERK-independent EGFR downstream targets, which ultimately influence the transformation process. Our data obtained in PTC patients, showing correlation of BRAF V600E not only with increased expression of MIG-6, but also with decreased activities of the EGFR and the PI3K/AKT pathways, further support this hypothesis and provide the basis for future studies specifically addressing this issue. These findings are also in agreement with previous studies, where activation of the EGFR was shown to facilitate transformation by mutant KRAS and oncogenic SOS with mutated KRAS being able to activate the EGFR by the induction of autocrine growth factors [53–55]. BRAF on the other hand induced the activation of an EGFR phosphatase, CDC25C, via the ERK pathway, which consequently inhibited EGFR phosphorylation [6]. Autocrine mechanisms for EGFR activation in tumors are commonly observed while alternative mechanisms, such as alteration of feedback loops, are only beginning to be discovered [56, 57]. Apart from the negative CDC25C feedback, SPRY2 and SOX10 were described as feedback inhibitors of the EGFR [6–8]. In agreement with Girotti et al. our data suggest that MIG-6 is a novel player within this process [27]. The fact that oncogenic BRAF signaling induces a feedback inhibitor of transformation indicates that a fine balance between promoting and inhibiting forces are required for optimal transformation, or that the induction of MIG-6 is a remnant of its original function as a tumor suppressor. Our data in PTC support the latter hypothesis, however, it has to be noted that specific mouse models will be needed to delineate whether MIG-6 truly inhibits BRAF induced tumorigenesis. Additionally, the fact that BRAF V600E downregulated EGFR activity via MIG-6 induction and that this effect could be reversed by inhibition of RAS-ERK signaling, allows for speculations about a potential role of MIG-6 as biomarker for resistance to drugs inhibiting RAF activity. Analysis of MIG-6 expression levels could thereby help to select patients most likely to benefit from combined anti-EGFR and anti-RAF approaches.

PTC is a cancer entity that has been associated with abnormal RAS-ERK signaling and is further characterized by a high frequency of BRAF V600E mutations [49, 58]. Recent studies suggested a potential tumor suppressor function of MIG-6 within this tumor entity, which was based on the analysis of primary patient samples on the one hand, as well as *in-vitro* and *in-vivo* studies on the other hand [17, 26, 48]. Although they only studied a small cohort of patients, Ruan and coworkers even suggested a BRAF V600E specific tumor suppressor function of MIG-6 in PTC, which was based on the observation that V600E mutated patients with high MIG-6 expression had a more favorable clinical course as those with low MIG-6 levels [26]. In our studies now, comprising data of almost 400 PTC patients, BRAF V600E correlated to the induction of *MIG-6* expression, which was accompanied by inactivation of both, EGFR and ERBB2. These data are in agreement with our *in-vitro* experiments and further strengthen the role of a MIG-6 mediated feedback from BRAF V600E to the EGFR family within this cancer entity. Interestingly, this MIG-6 mediated downregulation of the EGFR correlated to the inactivation of the PI3K/AKT pathway, another signaling module activated by the EGFR. This is of interest, as MIG-6 mediated inhibition of BRAF induced transformation is mediated neither via BRAF/MIG-6 interaction, nor via inhibition of the RAS-ERK pathway. As mentioned above, one might speculate that the inhibition of BRAF induced transformation by MIG-6 is rather mediated via the downregulation of the EGFR and other EGFR-mediated pathways, a hypothesis, which is currently elaborated in an ongoing project within our laboratory. In consideration of the data obtained within this study, the anti-apoptotic PI3K/AKT pathway is a good candidate, which is additionally supported by the fact that its inhibition is synergistic with RAF inhibitors to block proliferation in thyroid cancer cell lines [59].

Furthermore, low *MIG-6* expression levels correlated with a more aggressive phenotype of PTC in our analyses. Interestingly, this association was not apparent, when we analyzed the total cohort and BRAF WT patients. Only when we specifically focused on BRAF V600E mutated cases, low *MIG-6* expression was significantly associated with a high-risk stage. It has to be mentioned that these data need confirmation in prospective and randomized trials specifically addressing this issue. However, when the previous work within this field is taken into account, they support the hypothesis of a BRAF V600E specific inhibitory role within this tumor entity [26]. In this scenario, secondary (epi-) genetic alterations, causing downregulation of MIG-6 might be responsible for an increased BRAF oncogenic potential and the development of more aggressive tumors. Most interestingly, such a hypothesis is supported by recent findings of MIG-6 silencing in PTC in a substantial subset of PTC cases by promoter methylation [17]. Our own data, showing the absence of MIG-6 overexpression in BRAF V600E mutated high risk tumors, as well as the correlation between MIG-6 methylation and downregulation further support this assumption.

Taken together, we show that MIG-6 acts to restrain cellular transformation driven by oncogenic BRAF. Our results further indicate that mutant BRAF is able to induce a negative feedback circuit directed towards the EGFR, which in turn limits its own oncogenic potential, and that MIG-6 constitutes a key player within this process. The analysis of a cohort of primary PTC patient samples suggests that MIG-6 feedback inhibition of the EGFR is clinically relevant and may determine the prognosis of BRAF mutated PTC patients.

## Supporting Information

S1 File NIH3T3 Cells Respond to EGFR Inhibition. (DOCX)

S2 File MIG-6 Interacts with WT and Mutant BRAF in Human Cell Lines. (DOCX)

S3 File MIG-6 does not Affect BRAF Kinase Activity. (DOCX)

S4 File MIG-6 does not Affect BRAF/RAF1 Heterodimerization. (DOCX)

S5 File BRAF V600E induces MIG-6 Expression at a Transcriptional Level. (DOCX)

S6 File BRAF Inhibition by Vemurafenib decreases MIG-6 Expression in a BRAF V600E Mutated Cell Line. (DOCX)

S7 File BRAF V600E correlates to Activation of MEK1/2 and Inactivation of both EGFR and ERBB2 in Papillary Thyroid Cancer. (DOCX)

S8 File BRAF V600E correlates to Inactivation of the PI3K/AKT Pathway in Papillary Thyroid Cancer. (DOCX)

S9 File Methylation of MIG-6 correlates to its Downregulation in Papillary Thyroid Cancer. (DOCX)

S1 Table Primers used for qPCR. (PDF)
