# Supplementary material for: Mitogen-Inducible Gene-6 Mediates Feedback Inhibition from Mutated BRAF towards the Epidermal Growth Factor Receptor and Thereby Limits Malignant Transformation
Source: PLoS One. 2015 Jun 12;10(6):e0129859. doi: 10.1371/journal.pone.0129859 (PMC4466796; doi:10.1371/journal.pone.0129859)
Supplement: S1 File — (DOCX) [file pone.0129859.s001.docx]

**
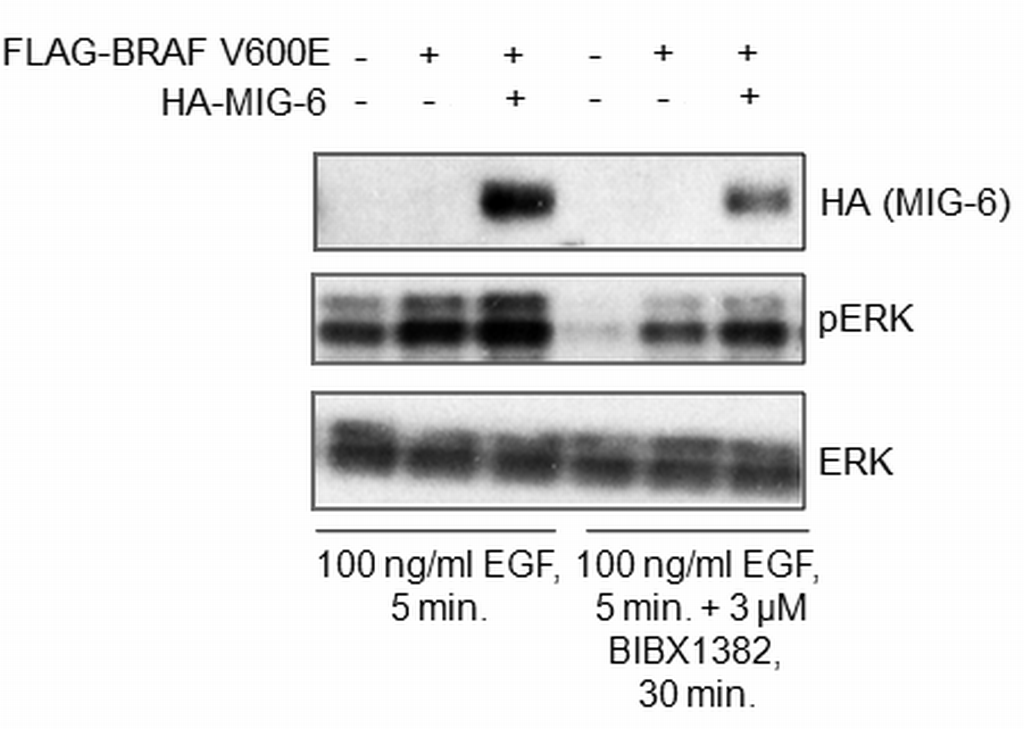
**

**S1 File. NIH3T3 Cells Respond to EGFR Inhibition.** NIH3T3 clone seven cells were transiently transfected with the indicated expression vectors. Additionally, cells were treated with 100ng/ml EGF for five minutes with and without simultaneous EGFR inhibition by BIBX1382 as indicated. The selectivity of BIBX1382 as a potent EGFR inhibitor was previously reported [1]. Whole cell extracts were immunoblotted with the indicated antibodies.

1. Solca FF, Baum A, Langkopf E, Dahmann G, Heider KH, Himmelsbach F, et al. Inhibition of epidermal growth factor receptor activity by two pyrimidopyrimidine derivatives. J Pharmacol Exp Ther. 2004;311: 502-509.
