# Supplementary material for: Mitogen-Inducible Gene-6 Mediates Feedback Inhibition from Mutated BRAF towards the Epidermal Growth Factor Receptor and Thereby Limits Malignant Transformation
Source: PLoS One. 2015 Jun 12;10(6):e0129859. doi: 10.1371/journal.pone.0129859 (PMC4466796; doi:10.1371/journal.pone.0129859)
Supplement: S2 File — (DOCX) [file pone.0129859.s002.docx]

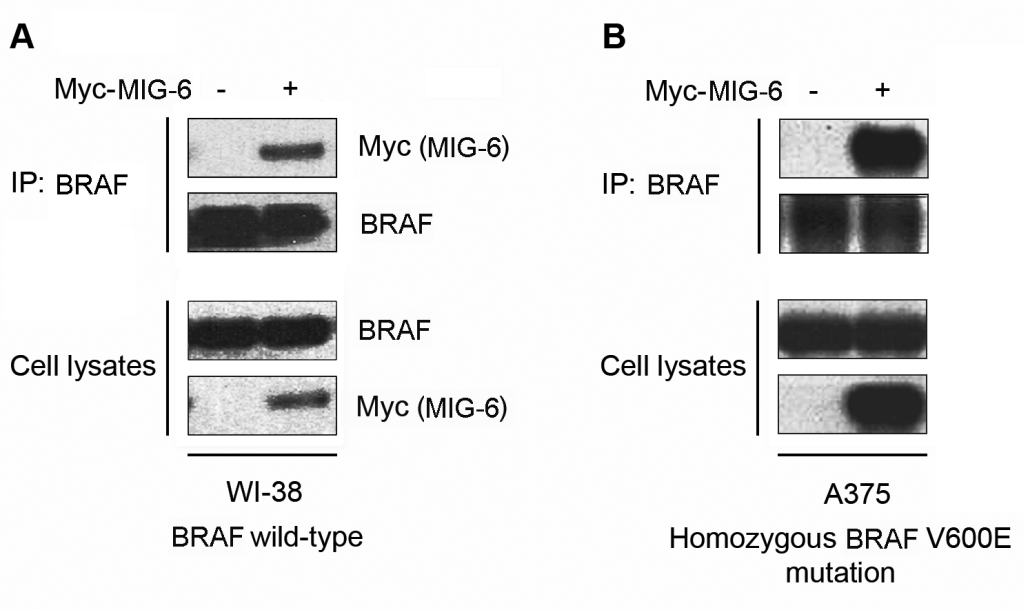


**S2 File. MIG-6 Interacts with WT and Mutant BRAF in Human Cell Lines. A.** WI-38 cells were transiently transfected with Myc-MIG-6 expression vector. Whole cell extracts were immunoprecipitated with anti-BRAF antibody followed by Western blotting and immunodetection with the indicated antibodies. **B.** A375 cells were transiently transfected with Myc-MIG-6 plasmid DNA. Cell extracts were purified with anti-BRAF IP and immunoblotted.
