# Supplementary material for: Mitogen-Inducible Gene-6 Mediates Feedback Inhibition from Mutated BRAF towards the Epidermal Growth Factor Receptor and Thereby Limits Malignant Transformation
Source: PLoS One. 2015 Jun 12;10(6):e0129859. doi: 10.1371/journal.pone.0129859 (PMC4466796; doi:10.1371/journal.pone.0129859)
Supplement: S3 File — (DOCX) [file pone.0129859.s003.docx]

**
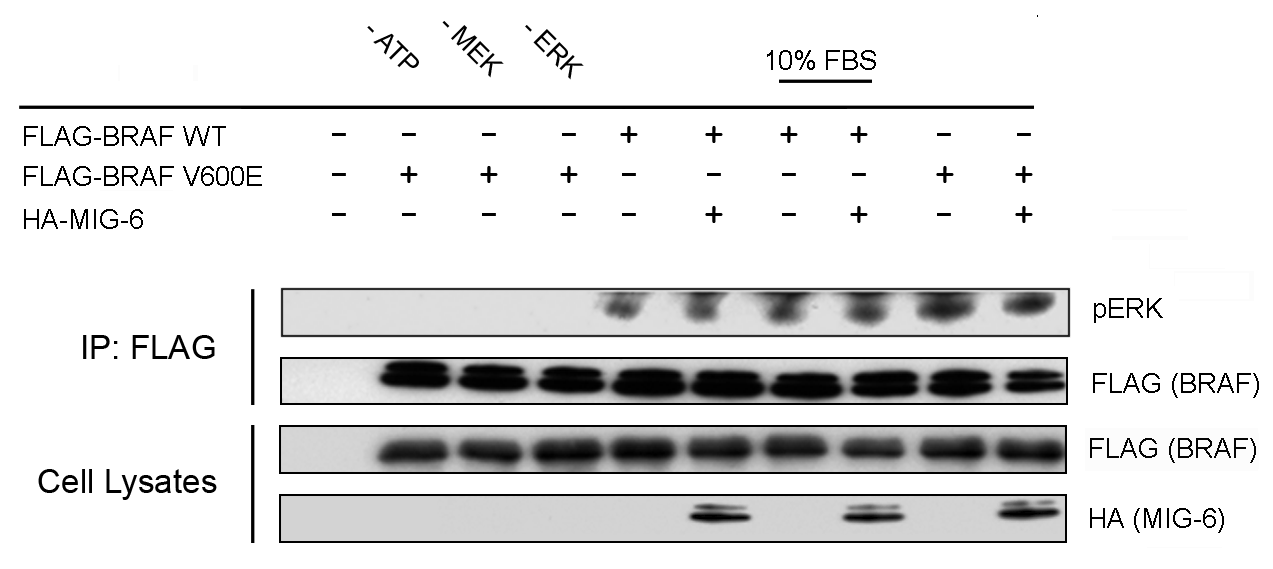
**

**S3 File. MIG-6 does not Affect BRAF Kinase Activity.** Cos-1 cells were transiently co-transfected with plasmids encoding FLAG-BRAF WT or FLAG-BRAF V600E and with HA-MIG-6 expression vector. Serum starved cells were stimulated with 10% FBS as indicated. Subsequently, total cell extracts were prepared and subjected to immunoprecipitation with anti-FLAG beads. Precipitates were washed, resuspended in kinase buffer and incubated with recombinant MEK1 and ERK proteins in the presence of ATP. A set of controls was included as depicted. Reactions were analyzed by Western blotting.
