# Supplementary material for: Mitogen-Inducible Gene-6 Mediates Feedback Inhibition from Mutated BRAF towards the Epidermal Growth Factor Receptor and Thereby Limits Malignant Transformation
Source: PLoS One. 2015 Jun 12;10(6):e0129859. doi: 10.1371/journal.pone.0129859 (PMC4466796; doi:10.1371/journal.pone.0129859)
Supplement: S4 File — (DOCX) [file pone.0129859.s004.docx]

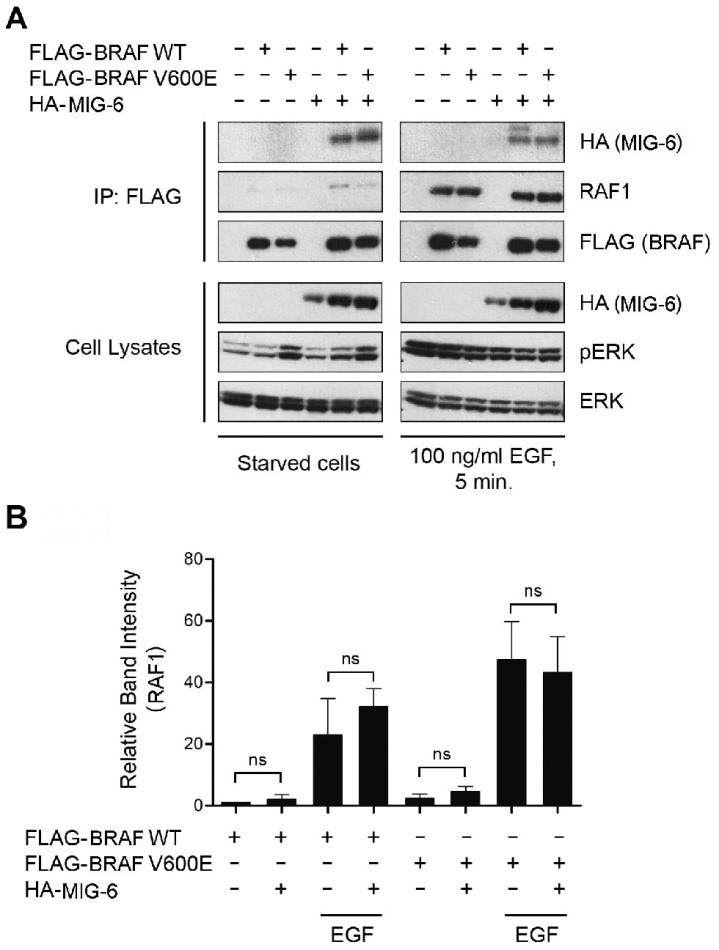


**S4 File. MIG-6 does not Affect BRAF/RAF1 Heterodimerization.** **A.** Cos-1 cells were transiently transfected with FLAG-tagged *BRAF* WT and V600E, respectively, either alone or in combination with plasmid DNA encoding *HA-MIG-6*. Serum starved cells were stimulated with 100ng/ml EGF for 5 minutes. Total cell extracts were subjected to anti-FLAG specific IP and analyzed by Western blotting. **B.** Densitometric quantification of immunoprecipitated RAF1 proteins normalized to the RAF1/FLAG-BRAF WT co-immunoprecipitation in starved cells. Graphs represent the mean of three independent experiments ± SD. Statistical significance was calculated using paired Student`s t-test. (ns, non-significant).
