# Supplementary material for: Mitogen-Inducible Gene-6 Mediates Feedback Inhibition from Mutated BRAF towards the Epidermal Growth Factor Receptor and Thereby Limits Malignant Transformation
Source: PLoS One. 2015 Jun 12;10(6):e0129859. doi: 10.1371/journal.pone.0129859 (PMC4466796; doi:10.1371/journal.pone.0129859)
Supplement: S5 File — (DOCX) [file pone.0129859.s005.docx]

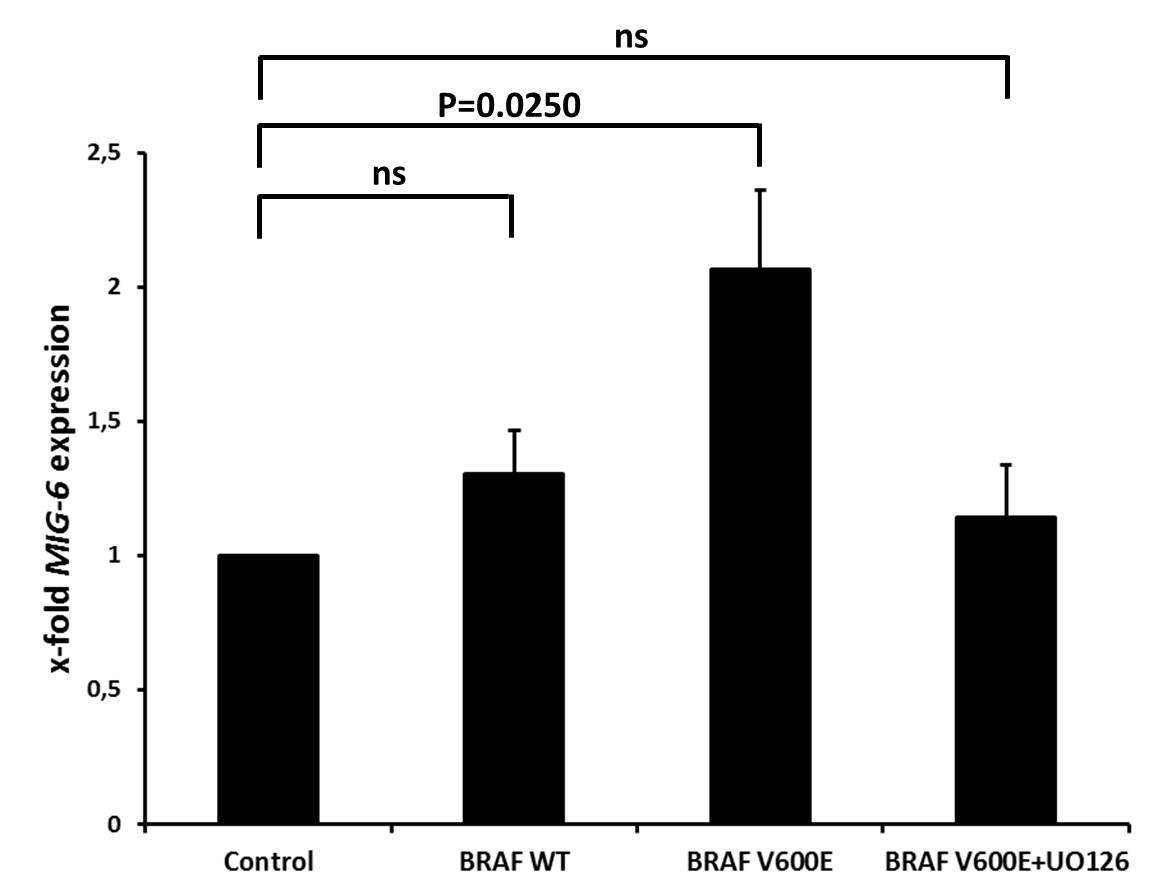


**S5 File. BRAF V600E induces MIG-6 Expression at a Transcriptional Level.** A431 cells were transfected with the indicated constructs and *MIG-6* mRNA expression was measured by qPCR. Graphs represent the mean of three independent experiments ± SD, expression values are given as x-fold expression of the calibrator (vector transfected control). Statistical significance was calculated using using paired Student`s t-test. (ns, non-significant). Different expression between the groups was additionally confirmed by analysis of variance (ANOVA; P=0.0008).
