# Supplementary material for: Mitogen-Inducible Gene-6 Mediates Feedback Inhibition from Mutated BRAF towards the Epidermal Growth Factor Receptor and Thereby Limits Malignant Transformation
Source: PLoS One. 2015 Jun 12;10(6):e0129859. doi: 10.1371/journal.pone.0129859 (PMC4466796; doi:10.1371/journal.pone.0129859)
Supplement: S6 File — (DOCX) [file pone.0129859.s006.docx]

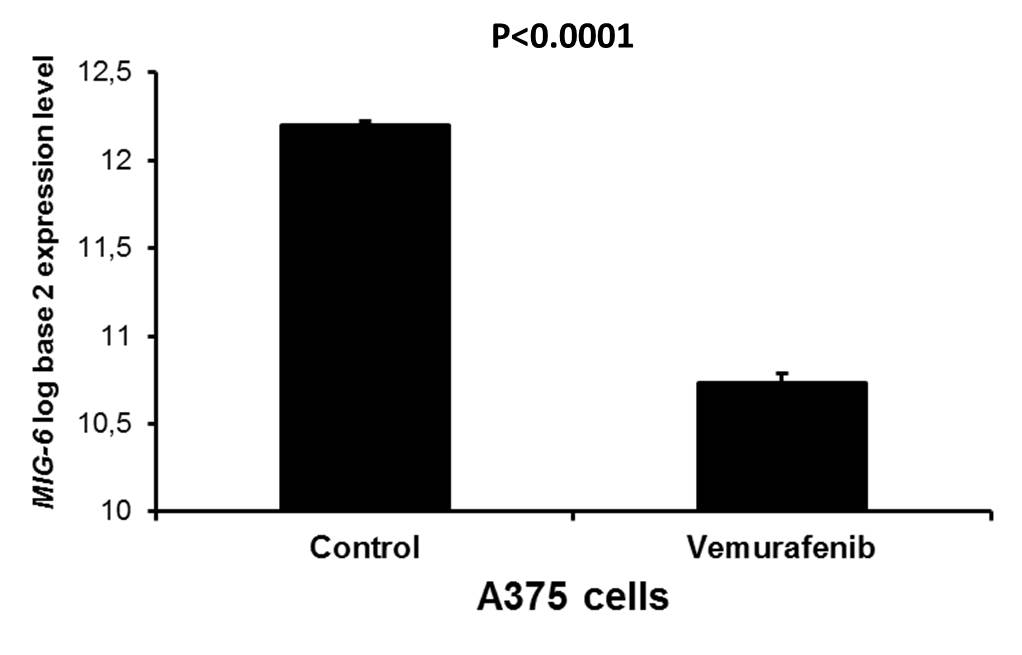


**S6 File. BRAF Inhibition by Vemurafenib decreases *MIG-6* Expression in a BRAF V600E Mutated Cell Line**. Parmenter and coworkers performed microarray expression analyses in the BRAF V600E mutated A375 melanoma cell line with and without treatment with vemurafenib, a specific BRAF inhibitor [1]. We analyzed *MIG-6* expression within this dataset via the Gene Expression Omnibus (<http://www.ncbi.nlm.nih.gov/geo/>), data were downloaded on 31 March 2015**.** Graphs represent the mean of three independent experiments ± SD. Statistical significance was calculated using Student`s t-test.

1. Parmenter TJ, Kleinschmidt M, Kinross KM, Bond ST, Li J, Kaadige MR, et al. Response of BRAF-mutant melanoma to BRAF inhibition is mediated by a network of transcriptional regulators of glycolysis. Cancer Discov.2014;4: 423-433.
