# Supplementary material for: Mitogen-Inducible Gene-6 Mediates Feedback Inhibition from Mutated BRAF towards the Epidermal Growth Factor Receptor and Thereby Limits Malignant Transformation
Source: PLoS One. 2015 Jun 12;10(6):e0129859. doi: 10.1371/journal.pone.0129859 (PMC4466796; doi:10.1371/journal.pone.0129859)
Supplement: S7 File — (DOCX) [file pone.0129859.s007.docx]

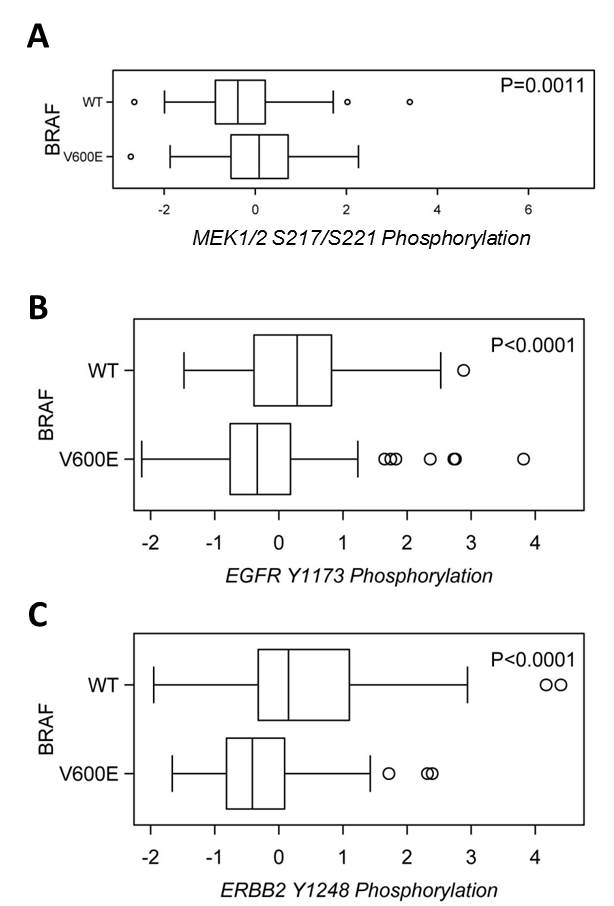


**S7 File.** **BRAF V600E correlates to Activation of MEK1/2 and Inactivation of both EGFR and ERBB2 in Papillary Thyroid Cancer.** Box plots showing a significant increase of MEK1/2 S217/S221 phosphorylation, as well as a significant decrease of EGFR Y1173 and ERBB2 Y1248 phosphorylation in papillary thyroid cancer (PTC) specimens carrying a BRAF V600E mutation. Data of PTC patient samples (n=197) were downloaded from the TCGA portal and analyzed using the cBioPortal for Cancer Genomics. The phosphorylation z-scores (displayed at the x-axis) were used for statistical calculation by the means of Mann-Whitney-Wilcoxon testing.
