# Supplementary material for: Mitogen-Inducible Gene-6 Mediates Feedback Inhibition from Mutated BRAF towards the Epidermal Growth Factor Receptor and Thereby Limits Malignant Transformation
Source: PLoS One. 2015 Jun 12;10(6):e0129859. doi: 10.1371/journal.pone.0129859 (PMC4466796; doi:10.1371/journal.pone.0129859)
Supplement: S9 File — (DOCX) [file pone.0129859.s009.docx]

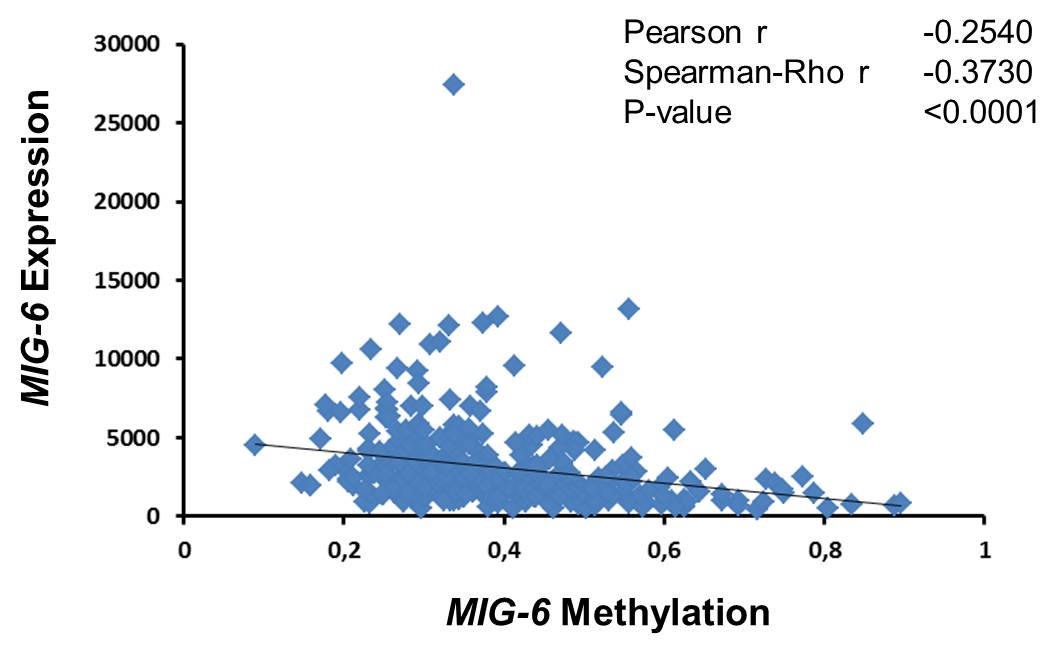


**S9 File.** **Methylation of MIG-6 correlates to its Downregulation in Papillary Thyroid Cancer.** Scatter plot showing a significant and inverse correlation between *MIG-6* expression (RSEM expression values displayed at the y-axis) and methylation (displayed as HM450 z-scores at the x-axis). Data of PTC patient samples (n=369) were downloaded from the TCGA portal, correlation coefficients and P-value was tested by the means of Spearman-Rho and Pearson analyses, respectively.
