# Supplementary material for: Mitogen-Inducible Gene-6 Mediates Feedback Inhibition from Mutated BRAF towards the Epidermal Growth Factor Receptor and Thereby Limits Malignant Transformation
Source: PLoS One. 2015 Jun 12;10(6):e0129859. doi: 10.1371/journal.pone.0129859 (PMC4466796; doi:10.1371/journal.pone.0129859)
Supplement: S1 Table — (PDF) [file pone.0129859.s010.pdf]

Gene Symbol	Gene name	Primer Sequence	Amplicon Size	PCR efficiency [%]
ERFFI 1 (MIG-6)	*ERBB Receptor Feedback Inhibitor 1 (Mitogen-Inducible Gene 6)*	fw: CTGGAGCAGTCGCAGTGAG<br>rev: GCCATTCATCGGAGCAGATTTG	136bp	96.47
GAPDH	*Glyceraldehyde-3-Phosphate Dehydrogenase*	fw: CAACAGCGACACCCACTCCT<br>rev: CACCCTGTTGCTGTAGCCAAA	115bp	101.34
RPL37A	*Ribosomal Protein L37A*	fw: ATTGAAATCAGCCAGCACGC<br>rev: AGGAACCACAGTGCCAGATCC	94bp	107.77%
GUSB	*Beta-Glucuronidase*	fw: GAAAATATGTGGTTGGAGAGCTCATT<br>rev: CCGAGTGAAGATCCCCTTTTTA	81bp	101.8
